# Effects of Temperature, Salinity, pH, and Light on Filtering and Grazing Rates of a Calanoid Copepod (Schmackeria dubia)

**DOI:** 10.1100/tsw.2008.153

**Published:** 2008-12-14

**Authors:** Changling Li, Xiaoxia Luo, Xianghu Huang, Binhe Gu

**Affiliations:** Department of Aquaculture, Fishery College, Guangdong Ocean University, 40 East Jiefang Road, Xiashan, Zhanjiang, Guangdong 524025, China

**Keywords:** copepods, environmental factors, grazing, filtering

## Abstract

Calanoid copepods are key components of the marine food web and the food sources of many larval fishes and planktivores, and grazers of phytoplankton. Understanding the ranges of major environmental variables suitable for their growth is essential to maintain the balance between trophic links and resources protection. In this study, the effects of temperature, salinity, pH, and light intensity on the filtering and grazing rates of a herbivorous copepod (*Schmackeria dubia*) were conducted in several control experiments. Our results indicated that experimental animals grazed normally at water temperatures between 15 and 35°C. The filtering and grazing rates increased by onefold at water temperatures from 15 to 25°C, with a peak at around 30°C. *S. dubia* fed normally at salinity ranging from 20 to 30 ppt, with significantly low filtering and grazing rates at salinity below 15 ppt and above 35 ppt. The filtering and grazing rates increased as pH increased, peaked at approximately 8.5, and then decreased substantially. Light intensity also displayed an important impact on the filtering and grazing rates. Filtering and grazing rates were high when light intensity was greater than 20 and less than 200 µmol m s. S. dubia nearly stopped feeding at low light intensity (less than 20 µmol m s).

### INTRODUCTION

Herbivorous copepods are the key components in the marine food web. They serve as the food sources for larval fishes[1,2] and grazers of planktonic algae[3,4]. Animal grazing (or top-down control of primary production) is viewed as a means to reduce algal biomass[4,5] and is influenced by animal size, food concentration, and other environmental variables. It is essential to obtain a better understanding of the environmental conditions for the growth of herbivorous marine copepods. The relationship between feeding rate and environmental variables has been studied in association with water temperature, food concentration, and animal size[3,6,7]. Very few studies have assessed the effects of pH and light intensity on copepod feeding ecology under laboratory conditions[8,9].

*Schmackeria dubia* is a commonly distributed copepod in the estuarine-coastal waters of the Taiwan Strait, South China Sea and the Indian Ocean[10]. It is a dominant copepod in the shrimp grow-out ponds in southern China[11]. *S. dubia* is a filter feeder with a body length normally ranging from 1 to 1.2 mm. This copepod is an excellent diet for larval fishes in commercial aquaculture[12] and could play a significant role in improving water quality by feeding on the intensive algal blooms in shrimp grow-out ponds. However, there have been no studies on the filtering and grazing rates, and the effects of major environmental variables on this copepod. This paper reports experiment results of the responses of the filtering and grazing rates by adult *S. dubia* to the wide ranges of water temperature, salinity, pH, and light. The purpose of this study was to provide insights into the major growth conditions of this copepod. Such information is essential to the protection, production as a larval feed, and the application of top-down control on algal production to improve water quality in costal aquaculture.

### MATERIALS AND METHODS

Experiments were conducted at Guangdong Ocean University located in Zhanjiang, China between June and August 2006.

#### Experimental Animals

*S. dubia* were collected from a shrimp aquaculture farm (water temperature, 30ºC; salinity, 27 ppt) in Zhanjiang, southern China. In the laboratory, the copepods were raised at a water temperature between 28 and ~30ºC, salinity of 27 ppt, and light intensity of 160–240 μmol $m^{-2}$ $s^{-1}$. After the copepods were acclimated to the culture condition, healthy energized egg-carrying females were carefully transferred with a pipe to the culture media. Their offspring were used as the experiment animals.

#### Food Source

A unicellular golden alga (*Isochrysis zhanjiangensis*), which has been used widely for the culture of larvae of various marine animals in southern China, was used as the food source. The alga was provided by the Algal Culture Research Laboratory of Guangdong Ocean University (Zhanjiang, China) and was grown using the Zhanshui 101 culture formula[13]. Algae at the exponential growth stage were used in our experiments.

#### Culture Media

Natural seawater was filtered using a plankton net with a fine mesh (200 μm) and boiled to kill all living organisms. Salinity gradient was made with distilled water and seawater. pH gradient was made by adjusting the media with diluted HCl or NaOH. During the experiments, pH was measured using a model MP220K bench pH meter and adjusted every 2 h to maintain the desired values. Clean wide-mouth transparent beakers (125 mL) were used as the culture containers.

#### Experiment Design

Healthy adult female copepods were starved for 24 h prior to experiments. A predetermined amount of the unicellular algal solution was added to each wide-mouth beaker containing 100 mL of seawater. Each treatment level consisted of eight replicates. One of the replicates was preserved with Lugol's iodine immediately and two replicates served as controls. Each of the remaining five replicates was stocked with five copepods. After 24 h, algal cells were preserved with Lugol's iodine and quantified using the Utermohl method.

There were five treatment levels for water temperature (15, 20, 25, 30, and 35ºC), seven for salinity (10, 15, 20, 25, 30, 35, and 40 ppt), five for pH (5.5, 6.5, 7.5, 8.5, and 9.5), and six for light intensity (2, 20, 80, 160, 200, and 240 µmol $m^{-2}$ $s^{-1}$). All experiments were conducted in Model FPG3 incubators (Ningbo Saifu Scientific, Ningbo, China). Each of these incubators contains three isolated compartments with adjustable temperature (±1.0°C), light density, and light:dark cycle.

The following environmental conditions were used during each experiment except when the variable served as an independent variable (see above): 28ºC as water temperature, 25 ppt as salinity, 8.5 as pH, and 160 µmol $m^{-2}$ $s^{-1}$ as light intensity. All experiments were conducted with an initial concentration of the unicellular alga at 20 × 104 cells $mL^{-1}$ and a 12:12-h light:dark cycle.

### Calculation of Filtering and Grazing Rates

The filtering and grazing rates of copepods were calculated using the following equations[14]:

$$F = \frac{V}{N} \times \frac{\ln C_1 - \ln C_2}{t} \quad (1)$$

$$G = F \times C = \frac{V}{N} \times \frac{\ln C_1 - \ln C_2}{t} \times \frac{C_0 - C_2}{\ln C_0 - \ln C_2} \quad (2)$$

where F is the filtering rate (mL $copepod^{-1}$ $h^{-1}$); G is the grazing rate (cells $copepod^{-1}$ $h^{-1}$); V is the volume (mL) of culture solution; N is the quantity of copepods in each experiment unit (bottle); $C_0$, $C_1$, and $C_2$ are the initial, control final, and replicate final algal density (cells $ml^{-1}$).

## RESULTS

Within the range of experimental water temperature (15–35ºC), copepods exhibited high filtering and grazing rates, which increased as water temperature increased. Both rates at 25 and 30ºC were twofold of those at 15ºC. After reaching a maximum at a water temperature between 25 and 35ºC, however, the filtering and grazing rates decreased, but were still higher than those at 15ºC (Fig. 1). The rate of increase slowed down when water temperature increased from 25 to 30ºC.

There were significant effects of salinity on the filtering and grazing rates of copepods (Fig. 2). Filtering and grazing rates were considerably lower at a salinity of 10 ppt than at other salinity levels, and increased rapidly when salinity was greater than 15 ppt. Both rates peaked between 20 and 30 ppt, and gradually decreased.

Both filtering and grazing rates increased linearly with pH from 5 to 8.5, with sharp declines when pH reached 9.5 (Fig. 3). Grazing rates at pH of 7.5 and 8.5 were 1.5- to 2.3-fold higher than those at a pH of 5.5 and 9.5 (Fig. 3). The optimal pH for both filtering and grazing rates fell between 7.5 and 8.5.

Significant effects of light intensity on copepod feeding were found during this study (Fig. 4). The filtering and grazing rates increased with light intensity, and reached their peaks when light intensity was greater than 80 and less than 200 µmol $m^{-2}$ $s^{-1}$. There were sharp decreases in filtering and grazing rates by copepods when light intensity reached 200 µmol $m^{-2}$ $s^{-1}$. Both filtering and grazing rates were minimal at the light intensity of 2 µmol $m^{-2}$ $s^{-1}$. Filtering and grazing rates at the light intensity of 160 µmol $m^{-2}$ $s^{-1}$ was 1.8-, 2.4-, and 3.6-fold higher than those at the light intensity of 240, 200, and 2 µmol $m^{-2}$ $s^{-1}$.

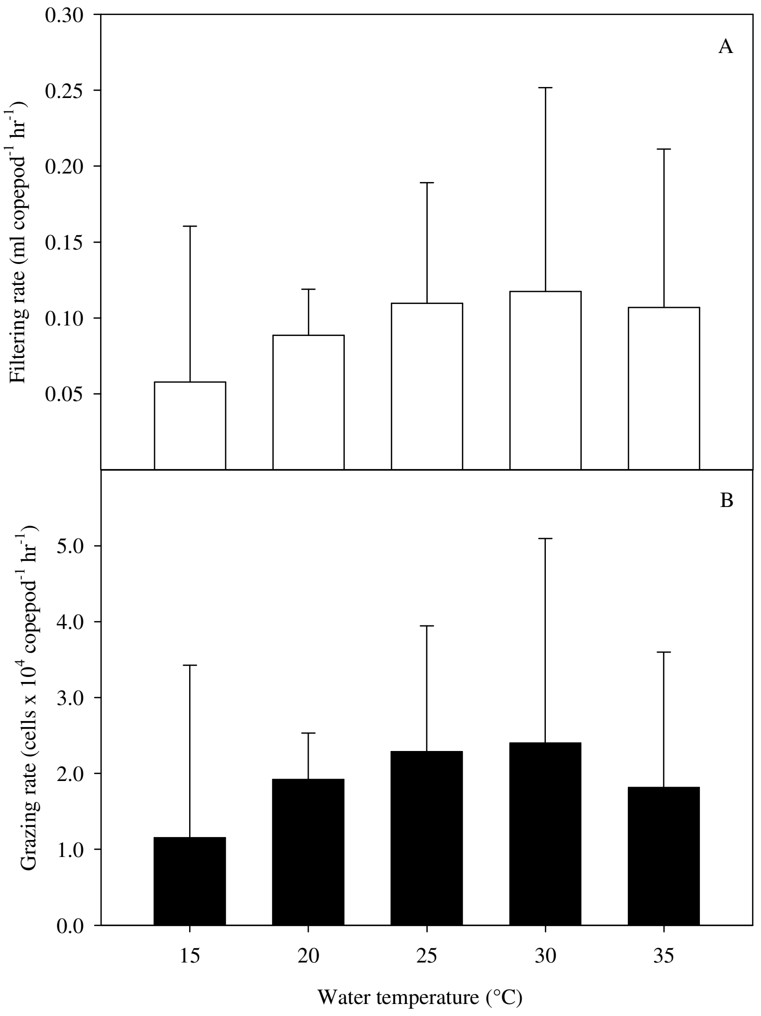

**FIGURE 1.** Plots of water temperature, filtering rate (A), and grazing rate (B) of a marine calanoid copepod (*S. dubia*). Error bars represent one standard deviation.

### DISCUSSION

The filtering rate by marine copepods has been studied intensively because it affects both animal growth and primary production[3,6,7]. Previous research has shown that the filtering rate increases with body size and food concentration up to some extent, then remains constant with further increases in food concentration[15]. The water volume filtered by a copepod ranges from >1.0 to 300 mL $d^{-1}$[16,17,18]. The average maximal filtering rate by *S. dubia* from all treatments was 2.5 mL $d^{-1}$, falling into the lower end of those previously reported. The ranges of water temperature, salinity, pH, and light intensity for the maximal filtering and grazing rates under our experimental conditions are listed in Table 1.

Water temperature affects respiration, reproduction, growth, development, and gut evacuation of marine copepods[19,20,21,22,23]. Filtering and grazing rates of copepods increase as water temperature increases within the normal range[24], but different species have different thermal regimes. For example, the subtropical copepod *Acartia pacifica Steue* feeds and grows well between 20 and 25ºC. Beyond 25ºC, both filtering and grazing rates decrease rapidly[25]. Lin et al.[26] reported a suitable water temperature range from 10 to 15ºC for a temperate species. Durbin and Durbin[27] showed that the maximal clearing rate by a marine copepod *Acartia hudsonica* at Narragansett Bay was similar at water temperatures between

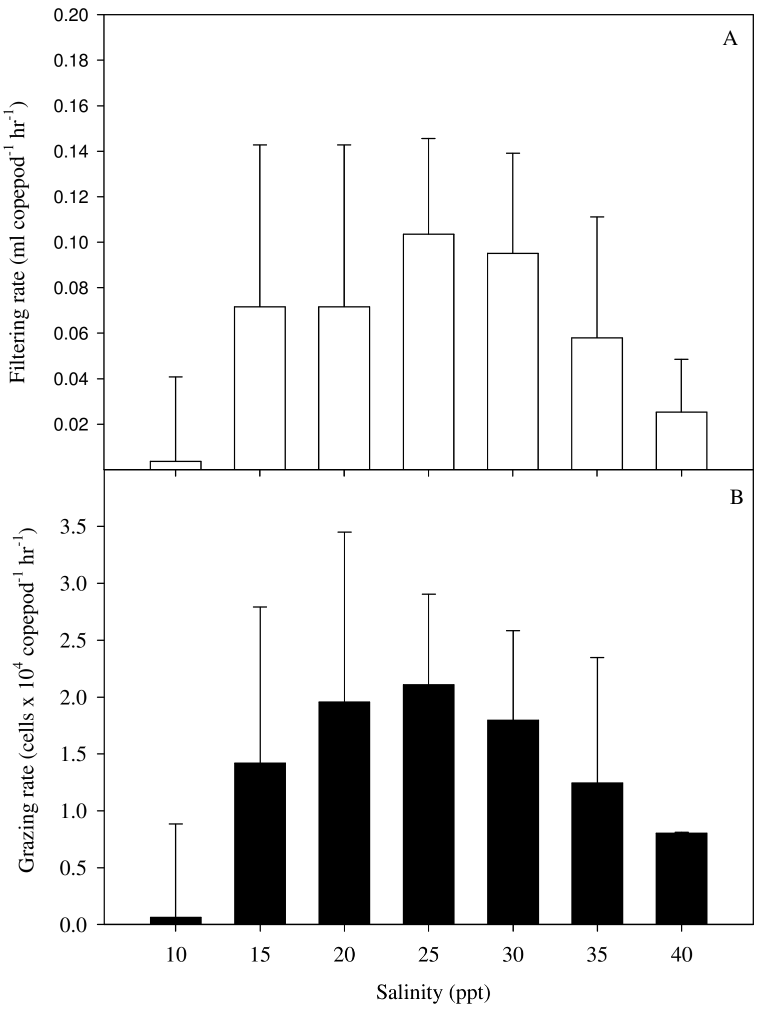

**FIGURE 2**. Plots of salinity on filtering rate (A) and grazing rate (B) of a marine calanoid copepod (*S. dubia*). Error bars represent one standard deviation.

4.5 and 16°C, with the maximal ingestion rate of the diatom *Thalassiosira constricta* at a water temperature of 16°C. Similar to this finding, our experiment indicated that *S. dubia* fed normally at water temperatures between 15 and 35ºC. This indicates that this species adapts to a broad range of water temperatures in the temperate to subtropical regions.

Research on the relationship between salinity and feeding behavior of copepods is rare. This is probably because salinity in the marine environment is relatively stable and copepods living in the estuarine area have adapted to the seasonal change in salinity. However, with increasing agricultural and domestic runoff to the coastal area in recent decades, it is important to understand the normal range of salinity for the estuarine species. Within the suitable salinity range, marine copepods are capable of regulating their osmotic balance and internal ionic concentration. Seuront[28] showed in a continuous culture that the complexity of the swimming paths of the males and nonovigerous females of an estuarine copepod (*Eurytemora affinis*) increased with the increase in salinity, while the ovigerous females became less mobile and sank. Lin et al.[26] reported that the filtering and grazing rates of a marine copepod (*Centropages mcmurrichi*) increased with the increase in salinity to 35 ppt and then decreased with further increases. Our study indicates that *S. dubia* can feed and grow well at salinity between 10 and 40 ppt with the optimal range from 20 to 30 ppt. This finding is in agreement with the estuarine habitat of *S. dubia*.

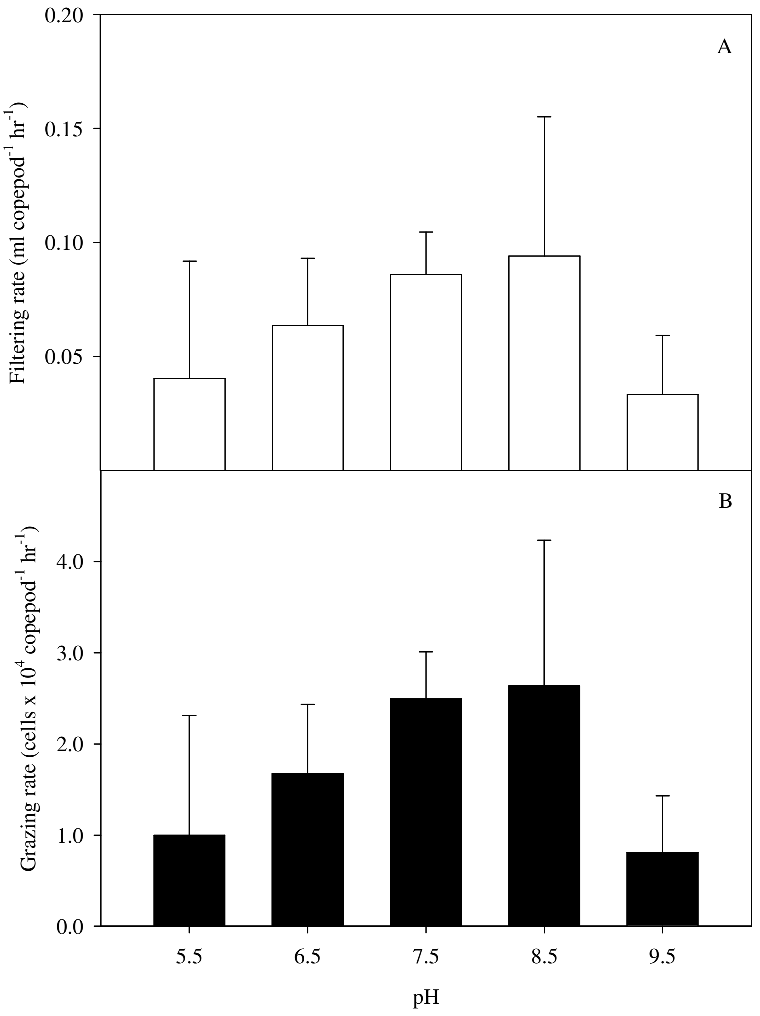

**FIGURE 3.** Plots of pH on filtering rate (A) and grazing rate (B) of a marine calanoid copepod (*S. dubia*). Error bars represent one standard deviation.

pH varies with aquatic acid-base balance. High pH may enhance the toxicity of some chemical compounds in the aquatic environment, thereby negatively affecting the health, feeding, and survival of marine copepods. However, little is known about the effects of pH on the physicoecology of marine plankton[8,9]. This is attributed to the view that pH in the marine environment is nearly constant (8.0 ± 0.5) due to the strong buffering capacity of the seawater[29]. This view might have changed recently because human-induced eutrophication leads to the frequent occurrence of phytoplankton blooms in coastal waters and high pH due to the photosynthetical removal of dissolved $CO_2$[30]. Pedersen and Hansen[8] found experimentally that natural plankton biomass increased within the pH range from 8 to 9. In the pH of 9.5 incubation, both phytoplankton and zooplankton biomass decreased. There is a lack of research on the response of copepods to the variations in ambient pH. Our study shows increases in filtering and grazing rates of *S. dubia* at pH from 5.5 to 8.5. Significant drops of both rates were found when pH reached 9.5, consistent with the finding from Pederson and Hansen[8].

Previous studies indicate that feeding by marine copepods usually takes place at night-time[31,32]. This observation has been confirmed with intestine pigment and enzymatic analysis[33,34,35]. However, Zhao et al.[36] reported a higher feeding rate of *Oithona similis* at day- than at night-time. Head et al.[37] indicated that two Arctic copepods (*Calanus hyperboreus* and *C. glacialis*) only fed when light intensity

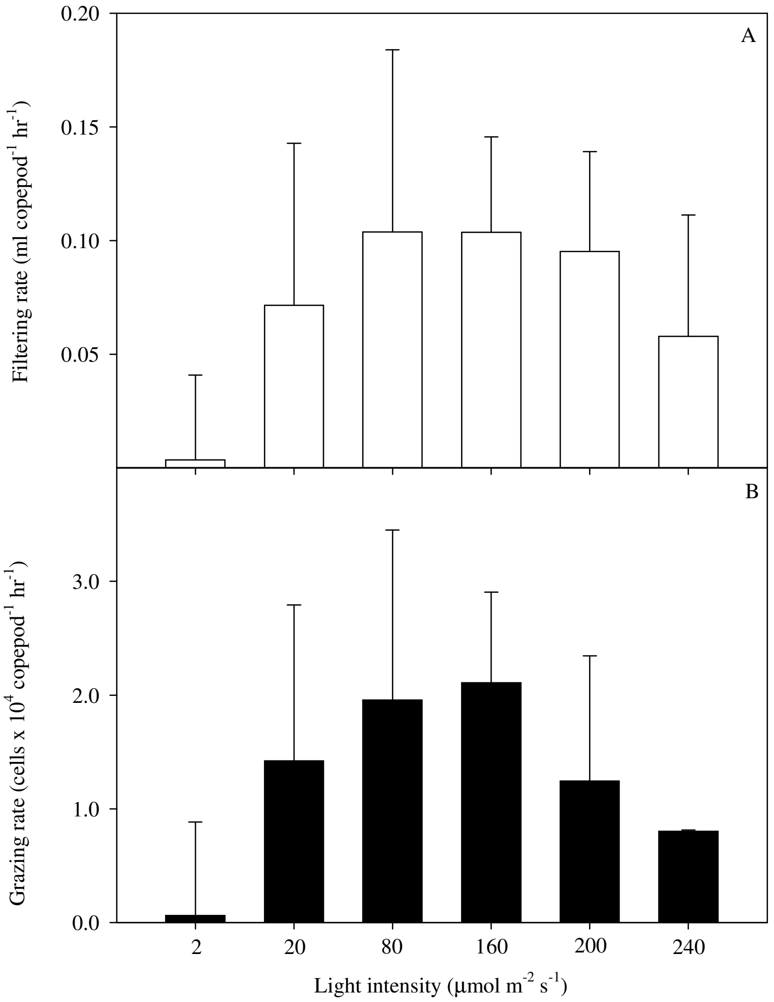

**FIGURE 4**. Plots of light intensity on filtering rate (A) and grazing rate (B) of a marine calanoid copepod (*S. dubia*). Error bars represent one standard deviation.

**TABLE 1**
**Ranges of Environmental Variables and their Corresponding Maximum Filtering and Grazing Rates of *S. dubia* in our Experiments**

| **Variable** | **Range** | **Filtering Rate (mL copepod$^{-1}$ h$^{-1}$)** | **Grazing Rate (cells x 10$^{4}$ copepod$^{-1}$ h$^{-1}$).** |
|---|---|---|---|
| Temperature (°C) | >25 and <35 | 0.12 | 2.40 |
| Salinity (ppt) | >20 and <30 | 0.10 | 2.11 |
| pH | >7.5 and <9.5 | 0.09 | 2.64 |
| Light (µmol m$^{-2}$ s$^{-1}$) | >20 and <200 | 0.13 | 3.99 |
| Average | | 0.11 | 2.79 |
| Standard deviation | | 0.02 | 0.83 |

decreased to <4 W $m^{-2}$ and stopped feeding in the morning. They speculated that the cessation of feeding during the daytime might be attributed to satiation. During a pre-experiment, we found no copepod feeding under 24-h continuous light regime. The current experiments indicated that peak feeding activities took place at a light intensity greater than 80 and less than 200 µmol $m^{-2}$ $s^{-1}$ under a 12:12-h light:dark cycle. There was probably no direct connection between light intensity and feeding activity. Increases in light intensity may promote algal photosynthesis and oxygen production, which in turn stimulate copepod feeding. However, further increases in light intensity resulted in the reduction of feeding activity. This might indicate that the exposure of copepods to extremely high light intensity is detrimental to their normal physiology. More research is needed to understand the relationship between feeding rates, light intensity, light cycle, medium oxygen production, and the physiological status of copepods.

This study characterized the ranges of water temperature, salinity, pH, and light level for the optimal filtering and grazing rates of a marine copepod under experimental conditions. Considering the recent anthropogenic impacts to coastal ecosystems, such information will provide useful insights into the living conditions of planktonic crustaceans that are critical not only to the protection of natural resources, but also to the study of food web functionality.
